# Abundance and *Leishmania* infection patterns of the sand fly *Psathyromyia cratifer* in Southern Mexico

**DOI:** 10.1371/journal.pntd.0012426

**Published:** 2024-09-10

**Authors:** Ana Celia Montes de Oca-Aguilar, Edith A. Fernández-Figueroa, Karina B. López-Ávila, Mariela Isabel Pavón-Méndez, Erika I. Sosa-Bibiano, Eduardo A. Rebollar-Téllez, Jorge A. Palacio-Vargas, Brenda García-López, Claudia Rangel-Escareño, Elsy Nalleli Loría-Cervera

**Affiliations:** 1 Immunology Laboratory, Regional Research Center “Dr. Hideyo Noguchi”, Autonomous University of Yucatan, Merida, Yucatan, Mexico; 2 Core B for Innovation in Precision Medicine, National Institute of Genomic Medicine, Mexico City, Mexico; 3 Medical Entomology Laboratory, Invertebrate Zoology Department; Faculty of Biological Sciences, Autonomous University of Nuevo Leon, San Nicolas de los Garza, Nuevo Leon, Mexico; 4 Directorate of Prevention and Health Protection of Health Services of the State of Yucatan, Merida, Mexico; 5 Population Genomics Department, National Institute of Genomic Medicine, Mexico City, Mexico; National Institutes of Health, UNITED STATES OF AMERICA

## Abstract

**Background:**

Localized cutaneous leishmaniasis (LCL) is a serious public health problem in Southern Mexico. Six species of Phlebotominae (Diptera: Psychodidae) have been found to be infected with *Leishmania (Leishmania) mexicana*, the causative agent of LCL in the region. However, little is known about the biology and potential participation of *Psathyromyia cratifer* in the *Leishmania* transmission cycle in Mexico, and the Americas. The present study provides evidence of temporal infection caused by *Leishmania* in *Psathyromyia cratifer* as well as data on its population dynamics in a LCL endemic area during the well-known transmission cycle of *Leishmania* in Southern Mexico.

**Methodology/Principal findings:**

Individual specimens of *Psathyromyia cratifer* were collected in four sites over the course of five months (from November 2020 through March 2021) using animal-baited, human-baited, and light traps. The temporal activity pattern (month + hour) of *Psathyromyia cratifer* was assessed along with its relationship with environmental variables. Moreover, *Leishmania* DNA and blood meals were analyzed and detected in female sand flies. This evidenced an infection rate ranging from 8% to 83%, and the record of *Homo sapiens* and *Ototylomys phyllotis* as blood hosts of this sand fly species. High abundances of these sand flies in human-baited traps were recorded which revealed the marked anthropophilic behavior of *Psathyromyia cratifer*. As regards the transmission dynamics of the parasite within the region, it was observed that the potential highest epidemiological risk for *Leishmania* transmission by *Psathyromyia cratifer* occurred during the months of January and March.

**Conclusion:**

This is the first contribution ever made to both the population dynamic and the temporal *Leishmani*a prevalence patterns in *Psathyromyia cratifer*. The resulting findings suggest that this sand fly specimen is the sixth potential vector of *L*. *(L*.*) mexicana* in Southern Mexico. Nonetheless, various biology, behavior, and ecology strands are yet to be addressed. The latter, to determine the role it plays in the transmission dynamics of the parasite within the region, and other areas of the country.

## Author summary

*Psathyromyia cratifer* is a species of Neotropical dipteran belonging to one of the most medically important groups worldwide wise, the Phlebotomine sand flies. Some sand fly species transmit *Leishmania* parasites to humans, which leads to one of the most common but neglected tropical diseases known as leishmaniasis. The data on life history traits and involvement of this species in the transmission of *Leishmania*, is yet limited. This research furnishes evidence about population dynamics and temporal *Leishmania* infection patterns in female *Pa*. *cratifer* specimens collected from four sites part of a transmission area in Yucatan, Mexico. Our findings suggest that females of this species are attracted to and feed on humans as well as an important *Leishmania* reservoir in the region. Although not all sites recorded infected individuals, the infection and temporal abundance of this species in most sites, were similar. Our findings suggest that this species could be a suspected vector of the parasite in the region, however, it is important to continue with incrimination studies that incorporate other sources of evidence.

## Introduction

Phlebotominae female sand flies (Diptera: Psychodidae) are hematophagous. This feeding behavior enables the interaction and transmission of a broad spectrum of pathogens causing disease in humans, such as leishmaniasis [1]. *Leishmania*, the etiological agent of this vector-borne disease, is an obligate intracellular parasite that naturally alternates between sand flies and wild vertebrates [2]. In humans, *Leishmania* infection varies in severity from asymptomatic to disfiguring cutaneous forms and fatal visceral disease [3]. These neglected diseases are worldwide severe public health problems in four eco-epidemiological regions of the world, where there is more than 300 million people living at risk of infection, around 0.9–1.6 million new cases, and about 20,000–40,000 deaths registered annually [4,5]. Consequently, vector incrimination studies represent the most crucial element for predicting transmission-expansion risks and designing preventive strategies in the case of leishmaniasis. Regardless of the preceding, the number of proven vectors that have shown to support the development of the parasite and its experimental transmission to a susceptible host in active transmission areas, is limited [6].

On the contrary, many sand fly species are considered potential vectors of this parasite when they are found to be infected by molecular methods, dissection of the digestive tract, or a combination of both [7,8]. In recent years, the molecular methods serving the detection of pathogens with high specificity and sensitivity, have greatly improved. *Leishmania* is no exception by turning the PCR (Polymerase Chain Reaction) and its different applications into an important tool for identifying a suspected vector. The latter represents a time-saving advantage when it comes about *Leishmania* typing [9–11]. This may be the first step to adopt prior to dissection method, which remains the most important stage for incrimination. Yet, it has several limitations as low rates of infection have been detected, and the species of the infecting *Leishmania* parasite cannot be determined [8,11,12].

Chiclero’s ulcer is the common name for localized cutaneous leishmaniasis (LCL) in Mexico. This disease generates ulcers that could rise to disfiguring lesions and mutilating scars that lead to disability, social stigma, and psychological repercussions [13]. The population at risk in Mexico is estimated to be 7.6 million people [14]. *Leishmania (L*.*) mexicana* is the species of parasite responsible for 99% of the LCL cases, and its first epidemiological and entomological research dates back to the early 20th century in the Yucatan Peninsula, a biogeographical province located in the southeast made up of the states of Campeche, Yucatan, and Quintana Roo [15].

The most outstanding advance in terms of vector incrimination in the Yucatan Peninsula, was achieved in 1965 when Dr. Francisco Biagi and his team demonstrated that *Bichromomyia olmeca olmeca* (Vargas & Díaz-Najera) was the proven vector of *L*. *(L*.*) mexicana* [16]. Since then, field studies have been conducted in the Yucatan Peninsula (YP) to disentangle the bionomics of vector species as well as to identify the epidemiological determinants of the *Leishmania* transmission cycle [17–19]. Current records from the region include 19 species, same that represent 39% of all sand fly species registered in Mexico hitherto [20]. These efforts have revealed that six species are participating in the transmission cycles of *L*. *(L*.*) mexicana* because they are highly abundant in the focus of LCL cases, present a marked anthropophilic profile and have been documented as naturally infected with the parasite [18–19,21–24]. However, most studies on incrimination are scarce and are concentrated in certain areas of the Peten Region, including the Campeche and Quintana Roo’s tropical rainforest. In the Peten region, the enzootic cycle of *L*. *(L*.*) mexicana* is seasonal as it occurs mainly after the rainy season, which correlates with the high abundance of sand fly vectors and reservoirs; and lasts five months (November-March) [17]. Most of the studies within the YP about vector incrimination have been conducted in this well-recognized period [21–23,25].

Although the first taxonomic record of Phlebotominae for Mexico occurred in Yucatan, this is the least explored geographic area regarding sand fly diversity, and vector incrimination. It is deemed that around 22–27 of these sand fly species may be present within the region [26]. Nevertheless, current records include 14 species deriving from sporadic sample collections done at the southern region [25,27] and two systematic studies developed in the east of the state [28,29]. Solely two of the three works on vector incrimination research in the state of Yucatan have been developed in already recognized foci near the endemic areas of Campeche [25,30]. Nonetheless, in 2015, the first description of an LCL emergent focus was made in a municipality located in the eastern part of the state, in an area with no previous record of the disease [31]. From there, around ten municipalities have documented human infection [32]. The two first integral eco-epidemiological studies on the determinants involved in the emergence of LCL in Yucatan showed a high abundance, anthropophilic profile, and prevalence infection of *Psathyromyia cratifer* (Fairchild & Hertig) [24,28,29] a sand fly species whose biology and participation in *Leishmania* transmission is little known.

*Psathyromyia (Psathyromyia) cratifer* is one of the 20 sand fly species that comprise the Shannoni series proposed by Fairchild (1965), and it was described from the collected specimens in Palenque, Chiapas, Mexico [33]. Since then, its presence has been recorded in the states of Veracruz, Campeche, Yucatan, and Quintana Roo [20,28]. Its distribution in the Neotropical region includes the Central American countries of Belize, Honduras, Costa Rica, and Panama [34], and it is likely to be present in Guatemala and the region of El Salvador. The biology and natural history of *Pa*. *cratifer* are yet unknown, and it has previously been suggested that *Pa*. *cratifer* is not an anthropophilic species [35,36]. Nevertheless, in the Yucatan Peninsula, high densities of *Pa*. *cratifer* and records of *Leishmania* infection have been reported in Shannon trap [24,29,37,38]. This study aims at providing data on the temporal pattern of natural infection by *L*. (*L*.) *mexicana* in *Pa*. *cratifer* for the very first time as well as to document its population dynamics and blood meal sources in four sites of a LCL focus on the state of Yucatan, Mexico.

## Materials and methods

*Ethics approval*.*-* This project was approved by the Ethics Research Committee of Regional Research Center “Dr. Hideyo Noguchi”, Autonomous University of Yucatan (Approval Number: CE-22-2018).

**Fig 1:**
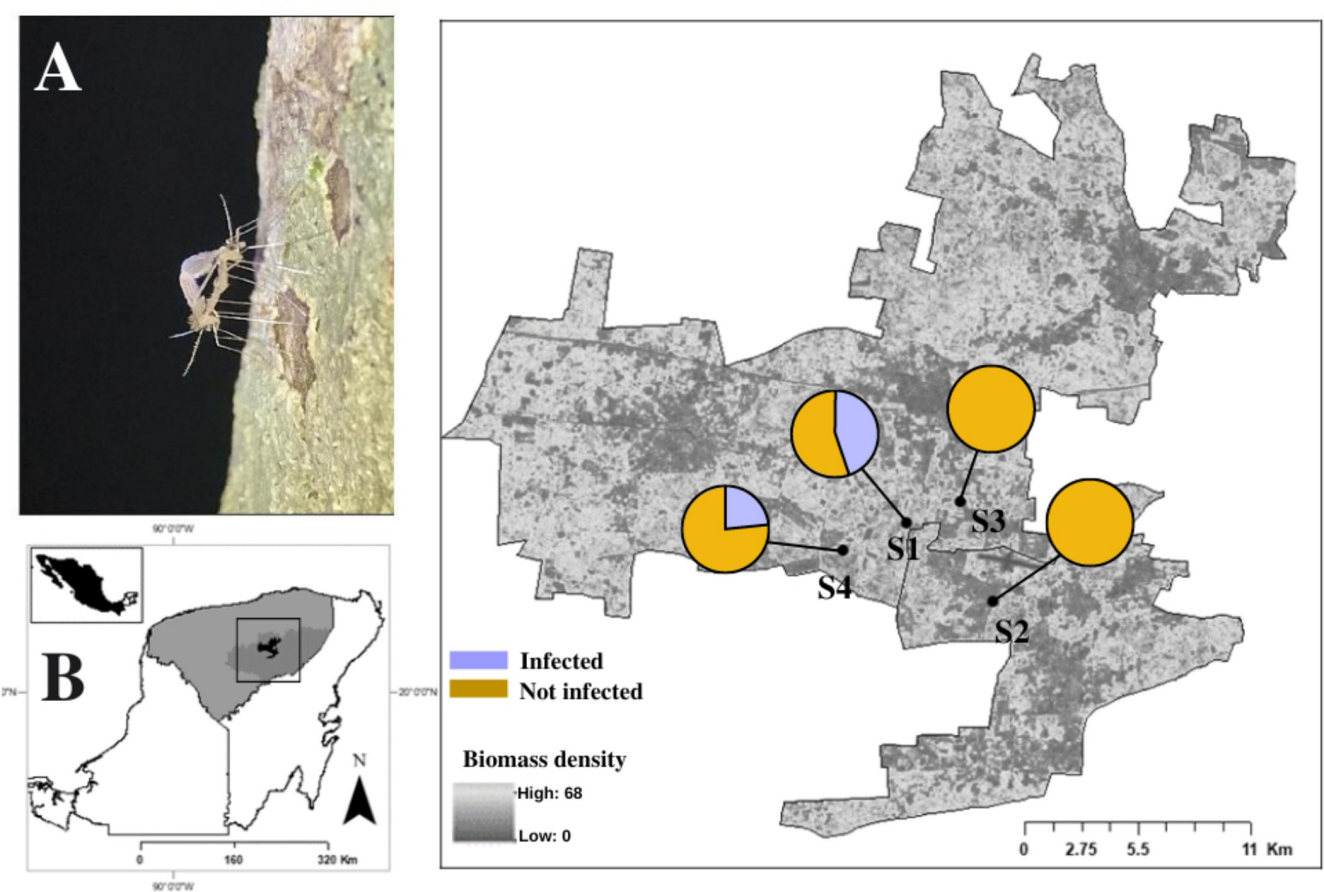
Sand fly collection localities in the study area and geographical distribution of the *Leishmania* prevalence in *Pa*. *cratifer* from a focus of localized cutaneous leishmaniasis (LCL) in Yucatan, Mexico. A) *Pa*. *cratifer* species during copulation (Photo: ACMOA) (https://www.inegi.org.mx/app/mapas/), B) Study area in Yucatan state, Mexico (https://en.www.inegi.org.mx/inegi/terminos.html); C) Spatial distribution by *L*. (*L*.) *mexicana* infection in *Pa*. *cratifer* in four sites with different forest cover expressed as biomass density (according to Alianza MREDD+, 2013). Site 1 (S1), Site 2 (S2), Site 3 (S3) and Site 4 (S4). The map was created using ArcGIS 10.3 (www.arcgis.com).

*Study Area*.*-* The study was conducted in an endemic area of LCL located in eastern Yucatan (Fig 1) from November 2020 through March 2021, same that has been previously recognized as the transmission period for *L*. *(L*.*) mexicana* in the region [17]. The climate in the area is mainly hot and humid (AW type) with an average annual temperature of 26.3°C, summer rains, and a dry winter [39]. The yearly rainfall reaches 89.9 mm. The predominant vegetation in this area used to be tropical dry forest. However, it has transformed through the years due to the happening of several rural activities related to agriculture and ecotourism. The floristic composition is characterized by the following dominant species: *Manilkara zapota* (L.) P. Royen, *Brossimm alicastrum* Swartz, *Bursera simaruba* (L.) Sarg, *Piscidia piscipula* (L.) Sarg, *Metopium brownei* (Jacq.) Urb and *Caesalpinia gaumeri* Greenm [40].

Four sites with an elevation <50 m and separated by a distance of 3 km served for the *Pa*. *cratifer* collection. All these sites have recorded human, wild rodent (*Heteromys gaumeri* Allen & Chapman and *Ototylomys phyllotis* Merriam) and *Pa*. *cratifer* infections by *L*. *(L*.*) mexicana* [24,31,41]. Site 1 is a fragment of preserved tropical dry forest of around 60 years located (20°39’32.5"N, 88°29’32.8"W) in the municipality of Tinum; site 2 is a fragment of vegetation in succession (“Acahual”) with 20 years of regeneration located at (20°39’32.50”N– 88°29’32.80”W) the municipality of Kaua; site 3 is an active “Milpa” (cultivation of corn-beans-squash) located (20°40’06.2”N, 88°28’08.30”W) in the village of Tohopku (Tinum). Site 4 (20°40’06.2”N– 88°31’12.7”W) corresponds to a secondary forest surrounding the X’calacoop (Tinum) village which is constantly impacted by local anthropic activities with successional variation records from 10 to 20 years.

*Sand fly collection and identification*.- *Pa*. *cratifer* specimens were gathered at each site for three consecutive nights during the well-recognized *Leishmania* transmission cycle for southern Mexico (November-March) by using eight CDC light traps (model 512; John W. Hock Company); eight Disney traps (baited with adults of *Peromyscus yucatanicus* Allen and Chapman), and a Shannon trap (baited with two persons) [42]. Samples of specimens were collected at different times for the first (12) consecutive days of each month. All the traps were active from 18:00 to 22:00 hours to estimate the period of hourly activity of *Pa*. *cratifer*. Except for the Shannon trap, the rest of the traps were placed in two 100 m transects and divided by a distance of 50 m. The traps were separated from each other in each transect by a distance of 30 m. A Shannon trap was placed at a distance of at least 80 m from the two transects. During the sand fly collection in the Shannon trap, temperature (T°C) and relative humidity (RH%) data were recorded every 20 minutes. Both variables showed significant differences between sampling sites and months (S1 Table). Therefore, this information was used in the subsequent analysis to evaluate the relationship at a fine scale. All sand fly specimens were collected by adhering to ethical standards approved by the Ethics Research Committee of Regional Research Center “Dr. Hideyo Noguchi”, Autonomous University of Yucatan (Approval Number: CEI-22-2018) in Merida, Yucatan.

Gathered sand flies were anesthetized with chloroform and preserved in 95% ethanol. Specimens were processed following the method proposed by Ibáñez-Bernal [42,43]. The head and abdominal segments VII-X of the female specimens were used for taxonomic identification considering the keys of Ibáñez-Bernal [42,43], while the rest of the body was used for the detection and identification of the *Leishmania* parasite.

*DNA extraction and detection of L*. *(L*.*) mexicana*.- Total genomic DNA was extracted individually from specimens collected in the Shannon trap using 250 μL of a 10% solution of the resin Chelex-100 [44], and 25 μL of proteinase K (INVITROGEN). Then, the samples were incubated at 56°C for 12 hours in a water bath and later subjected to 100°C for 15 minutes. The concentration of the extracted DNA from each specimen of *Pa*. *cratifer* was determined through a NanoDrop spectrophotometer (Thermo Scientific). The integrity and quality of each sample was verified by using PCR amplification of the constitutive gene COI (cytochrome oxidase subunit I). The latter allowed us to discard samples whose DNA quality was inadequate for the subsequent analysis to detect the *Leishmania* parasite, avoiding underestimating the prevalence in this way. To amplify ≈379–450 bp fragment of the COI gene, the primers L6655 (5’-CCG GAT CCT TYT GRT TYT TYG GNC AYC C-3’) and H7005 (CCG GAT CCA CAN CRT ART ANG TRT CRT G-3’) were used [45,46]. The commercial kit Taq PCR Master Mix (QIAGEN) was used for the amplification, and the reaction mix was prepared in a final volume of 25 μL using 12.5 μL of the master mix, 0.2 μM of each primer, 5.5 μL of nuclease-free water and 5 μL of DNA. The PCR reaction was done in a SimpliAmp thermal cycler (Applied Biosystems) under the following conditions: initial denaturation step at 95°C for 5 minutes, followed by 40 cycles of 92°C for 30 seconds, 45°C for 40 seconds, 65°C for 1.5 minutes. All this, with a final extension at 72°C for 10 minutes.

The nuclear ribosomal Internal Transcribed Spacer (ITS-1) was amplified to detect *Leishmania* DNA. Primers L5.8s (5’-TGA TAC CAC TTA TCG CAC TT-3’) and LITSR (5’-CTG GAT CAT TTT CCG ATG-3’) that amplify a fragment of ≈300–350 bp were used [47, 48]. The commercial kit Taq PCR Master Mix (QIAGEN) was used following the manufacturer’s instructions. PCR reactions were run in a final volume of 20 μL in a SimpliAmp thermal cycler (Applied Biosystem) under the following conditions: initial denaturation at 94°C for 4 minutes, 36 cycles at 94°C for 40 seconds, 54°C for 30 seconds, 72°C for 1 minute; and a final extension at 72°C for 6 minutes. DNA extracted from 5x10^7^ promastigotes of the MHET/MX/97/Hd18 strain of *L*. *(L*.*) mexicana* was used as a positive control using 1 ng of sample.

For *Leishmania* species typification, PCR products of *Pa*. *cratifer* specimens positive to *Leishmania* were re-amplificated with ITS-1 primers as was described, using 2 μL of the sample. The PCR products were subjected to digestion with Hae III restriction enzyme (Promega- USA) for *Leishmania* species identification. To this end, a mixture with 2 μL of nuclease-free water, 2 μL of buffer C 10X and, 1 μL of *Hae* III (10 U/μL) was added to 15 μL of the amplified product. Later, the microtube was incubated at 37°C for 3 hours and at 80°C for 20 minutes to inactivate the enzyme. The species profiles of each sample and reference controls were observed in a 4% agarose gel subjected to electrophoresis for 3 hours and stained with SYBR Gold Nucleic Acid Gel Stain (S11494, Invitrogen, USA). Four *Leishmania* species were used as positive controls: *L*. *(L*.*) mexicana* (L. m., MHOM/MX/2011/Lacandona)), *L*. *(L*.*) amazonensis* (L. a., MHOM/BR/1973/M2269)), *L*. *(L*.*) infantum* (L. i., MHOM/BR/72/BH46)) and *L*. *(Viannia) braziliensis* (L. b., MHOM/BR/1995/M15280). For the molecular identification of *Leishmania* infection, all samples were analyzed in two different laboratories (Immunology Laboratory, Regional Research Center “Dr. Hideyo Noguchi”, and Nucleus B for Innovation in Precision Medicine, National Institute of Genomic Medicine, INMEGEN) to avoid bias in the results obtained. The foregoing, given the large number of samples that were examined.

*Identification of blood meals*.- A blood feeding analysis was performed on *Pa*. *cratifer* females infected with *Leishmania*. Genomic DNA was used as a template in a conventional PCR using the following primers (FWD 5´CGA AGC TTG ATA TGA AAA ACC ATC GTT G 3´ and RVS 5´ TGT AGT TRT CWG GGT CHC CTA 3´) [49] to amplify a cytochrome B (c*ytB*) sequence. For the amplification, the commercial kit Taq PCR Master Mix (QIAGEN) was used, and the reaction mix was prepared in a final volume of 50 μL using 25 μL of the master mix, 2 μL of each primer (10 μM), 18 μL of nuclease-free water and 3 μL of DNA. The PCR reaction was done in a SimpliAmp thermal cycler (Applied Biosystems) under the following conditions: initial denaturation step at 95°C for 10 minutes, followed by 36 cycles of 95°C for 30 seconds, 55°C for 40 seconds, 72°C for 50 seconds; with a final extension at 72°C for 10 minutes. The PCR products were observed in a 2% agarose gel subjected to electrophoresis for 1 hour and stained with SYBR Gold Nucleic Acid Gel Stain (S11494, Invitrogen, USA). The PCR products were purified using the Agencourt AMPure XP kit (Cat. A63882, Beckman Coulter, Brea, CA, USA). Amplicons were sequenced using the BigDye Terminator v3.1 Cycle Sequencing Kit (Cat. 4337455, Thermo Fisher, Waltham, MA, USA). The samples were purified by the BigDye XTerminator (Cat. 4376486, Thermo Fisher, Waltham, MA, USA) before loading on the ABI 3730xL DNA analyzer (Thermo Fisher, Waltham, MA, US). Chromatograms were visualized, and sequences were edited manually and ensembled using BioEdit software (version 7.0.5). A consensus sequence was compared to reference sequences deposited in GenBank using the BLASTn 2.2.19. Species level identification was determined when sequences exhibited ≥ 93% identity. The sequences obtained in this study were deposited at GenBank (S5 Table).

*Statistical analysis*.*-* Data analyses were performed for *Pa*. *cratifer* according to a) abundance (obtained for the three traps used) and b) temporal infection rates in an endemic area of leishmaniasis. Generalized Linear Mixed Models (GLMM) assuming a negative binomial distribution (NB) were used to assess the spatial and temporal dynamics of *Pa*. *cratifer* at four sites within the LCL focus in Yucatan. The negative binomial distribution was selected as it better reflected the probability distribution of abundance. First, differences were assessed in abundance between sites where both, trap type (CDC and Shannon traps) and month were considered as random variables. Secondly, differences in abundance between months were evaluated where the type of trap was considered a random variable to determine the temporal trends of this sand fly species by each site. On the other hand, the catch data derived from the Shannon trap enabled us to assess the pattern of hourly activity (18:00–22:00 hours) where the site and the month were considered as random variables. For each GLMM, the goodness of fit by deviance and Akaike information criterion (AIC) were documented. When the model was significant, Tukey’s test was used with Bonferroni´s adjustment (differences in abundance among sites n = 6, temporal trend n = 10, hourly activity n = 10) in order to identify specific differences in abundance. An NB was also conducted to determine the influence of temperature and humidity on the activity patterns of this species (log10(Abundance)) at global, and local scales.

The prevalence of infection by *Leishmania* in specimens of *Pa*. *cratifer* was estimated by site and month. The Clopper-Pearson test was used to estimate their 95% confidence intervals (CI) and Fisher’s exact test to evaluate any associations between *Leishmania* infection, and site and month [50, 51]. Simulate P values were estimated with 2, 500 replicates using the Monte Carlo test and then we calculated a pairwise comparison to identify the specific association in prevalence.

All the analyses were performed with R v. 4. 2. 1. All the models were fitted using MASS packages [52] and lme4 [53]. Package multicomp [54] was employed for multiple comparisons. Packages GenBinomApps [55] and stat [56] were used to estimate the 95% IC and the association with *Leishmania* infection, respectively. Package companion [57] was employed for multiple comparisons using Fisher´s exact approach.

## Results

### Spatial and temporal fluctuation of the abundance of *Pa*. *cratifer* and their relationship with environmental variables

With a sample effort of 1, 440 night-traps, a total of 3,065 specimens of *Pa*. *cratifer* were collected in the study area. Overall, 94% of the females were collected in the human baited trap and only 2% (23/1404) and 0.5% (7/1404) were documented fed and gravid (presence of eggs), respectively. The Shannon trap caught 91% (2,795/3,065) of *Pa*. *cratifer* specimens, while CDC and Disney traps only caught 9% and 0.10%, respectively. Both sexes were equally caught with the Shannon’s trap (1404♀/1391♂~ 50%), while male were most dominant in CDC traps (♂67%/179; ♀33%/88) and Disney traps (♂67%/2; ♀33%/88). No statistically significant differences were found in the abundance of males (51%/ 1, 5723) and females (49%/1, 493) (χ^2^ = 0.001, Df = 1, p = 0.97). All fed females were captured in the Shannon trap. The number of females fed per site ranged from 1 to 10% (Table 1).

**Table 1:** Number of *Pa*. *cratifer* collected with three different traps in four sites from an emergent focus of LCL in Yucatan, Mexico. Shannon trap (Sh), CDC trap (CDC), and Disney trap (Dy). In parentheses, the number and percentage of females fed are shown. Sites with different superscript letters (a-c) mean a significant difference in abundance (p<0.001) according to the Tukey post hoc test.

Sites	Sh			CDC			Dy			TOTAL	Total Feed
	M	F	Feed	M	F	Feed	M	F	Feed		
S1	1303	1252	(8/1%)	156	70	(0/0%)	2	1	(0/0%)	2784^**d**^	(1/1%)
S2	5	10	(1/10%)	4	2	(0/0%)	-		(0/0%)	21^**a**^	(1/8%)
S3	20	37	(2/5%)	5	5	(0/0%)	-	-	(0/0%)	67^**b**^	(2/5%)
S4	63	105	(2/2%)	14	11	(0/0%)	-	-	(0/0%)	193^**c**^	(2/2%)
**Total Abundance**	1391	1404	(23/2%)	179	88	(0/0%)	2	1	(0/0%)	3065	(23/2%)

A spatial variation in the abundance of *Pa*. *cratifer (*χ^2^ = 523, Df = 3, p<2e-16) was recorded but there were no significant interactions between site and sex (Df = 3, χ^2^ = 3.71, p = 0.29). Many *Pa*. *cratifer* was collected from Site 1 and in the Shannon trap (Table 1). The observed abundance of *Pa*. *cratifer* was significantly lower in the rest of the sites (Table 1).

**Fig 2:**
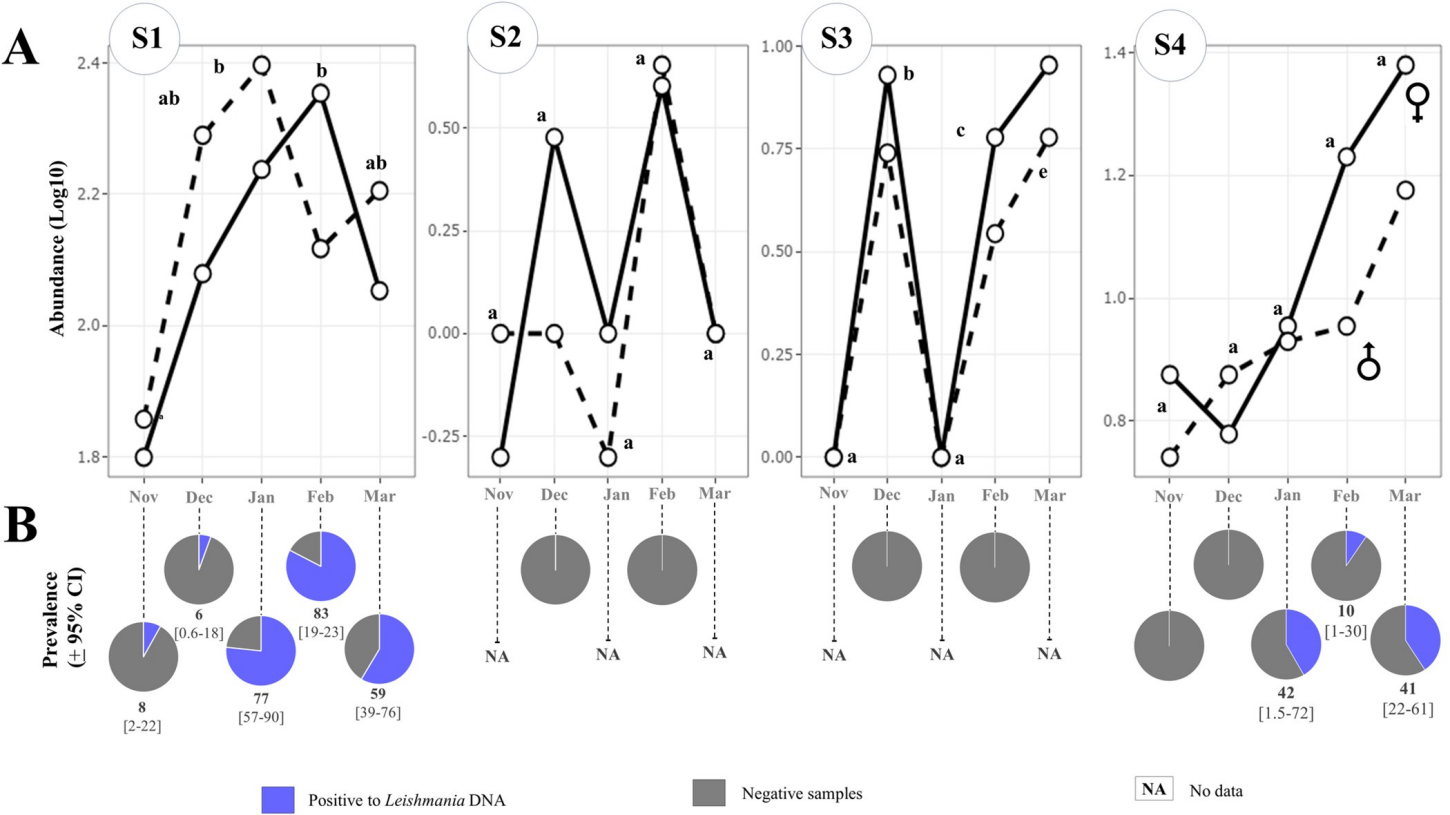
Temporal fluctuation of the abundance of *Pa*. *cratifer* (A) and *Leishmania* prevalence (B) in specimens collected in four sites during the LCL transmission cycle recognized in Yucatan. A) Total abundance (log10 transformed) of *Pa*. *cratifer* per month by sex considering the three captured methods (Shannon, CDC and Disney traps). Months with the same letter did not present significant differences (p<0.05) according to the Tukey post hoc test (a-e). B) *Leishmania* prevalence and 95% confidence interval in brackets (CI). Site 1 (S1), Site 2 (S2), Site 3 (S3), and Site 4 (S4). Female (F), Male (M).

Two of the four sites showed a significant trend in the temporal fluctuation of *Pa*. *cratifer*. However, in all sites, significant interaction effects were not found between month and sex (S2 Table). Overall, in this leishmaniasis focus, the period of greatest abundance of *Pa*. *cratifer* occurred from January to March (Fig 2). In sites 1 and 2, the abundance of *Pa*. *cratifer* was significantly different between months. In S1, the highest abundance was recorded in January and February, while in S3, a high record of this sand fly was documented in March, followed by December and February (Fig 2). Although no significant differences between months were detected at sites 2 and 4, peaks of high activity were observed in February and March, respectively.

**Fig 3:**
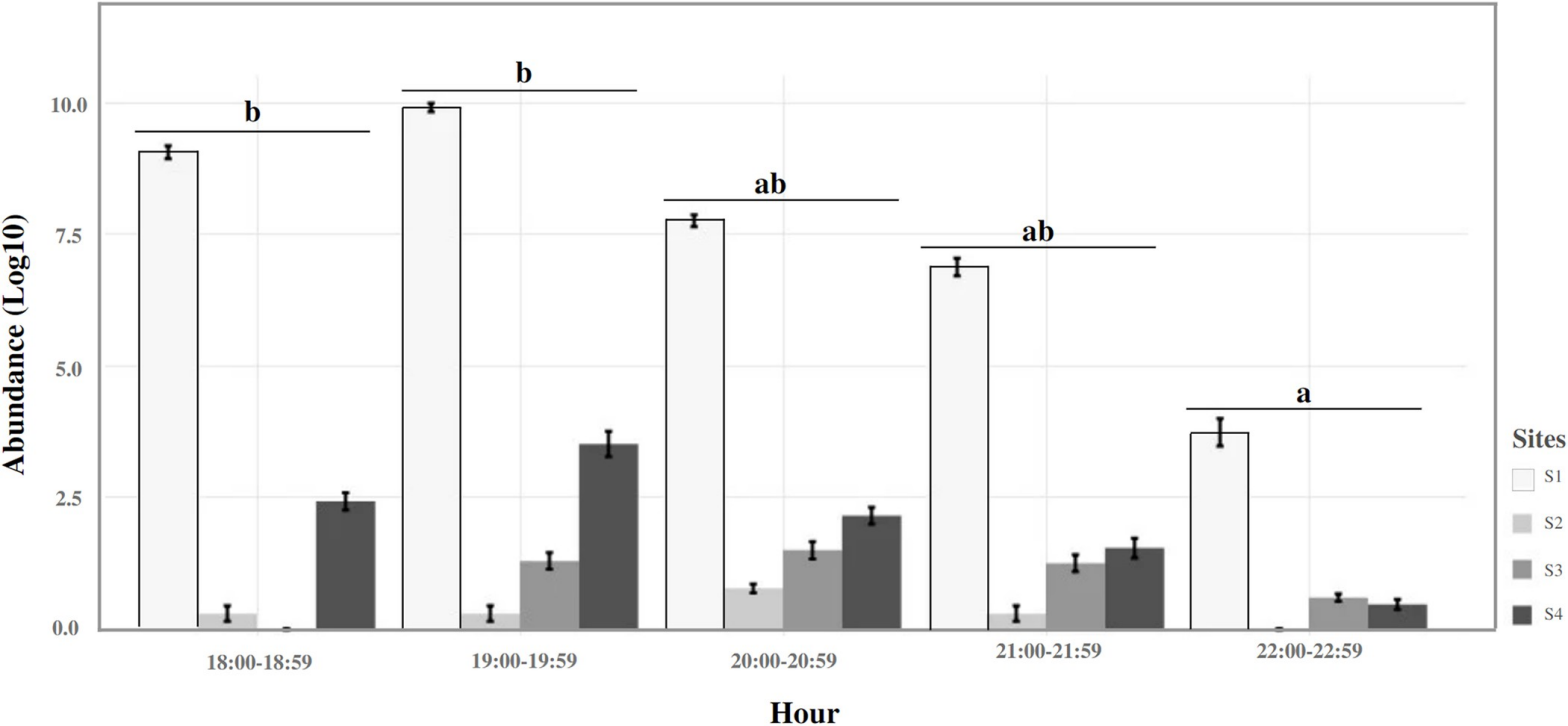
Hourly activity pattern (Abundance, ± standard error) of *Pa*. *cratifer* at four sites from an LCL focus in Yucatan, Mexico. The hours with the same letters do not represent significant differences in abundance according to the Tukey post hoc test.

The result of the generalized mixed model showed statistically significant dissimilarities in the hourly activity pattern (χ^2^ = 20.92, Df = 4, p = 0.0003). According to the post hoc test, the activity of *Pa*. *cratifer* in this focus of LCL increases significantly in the first two hours (18:00–19:59 h), showing slightly greater activity at 19:00–19:59 h. Although this general pattern was observed at sites S1 and S4 (Fig 3) where the abundance of this species was significantly high, at sites S2 and S3 the activity increased during the third hour of collection (20:00–20:50 hours, Figs 3 and S1).

Despite the temporal variations in the abundance of *Pa*. *cratifer*, the results of the generalized mixed model with negative binomial distribution showed that at general and local scales, the temperature (χ^2^ = 0.16, Df = 1, p = 0.68) and relative humidity (χ^2^ = 0.09, Df = 1, p = 0.76) were not related to the abundance of this sand fly species (S3 Table).

### Spatiotemporal distribution of *Leishmania* infection in *Pa*. *cratifer* females

**Fig 4:**
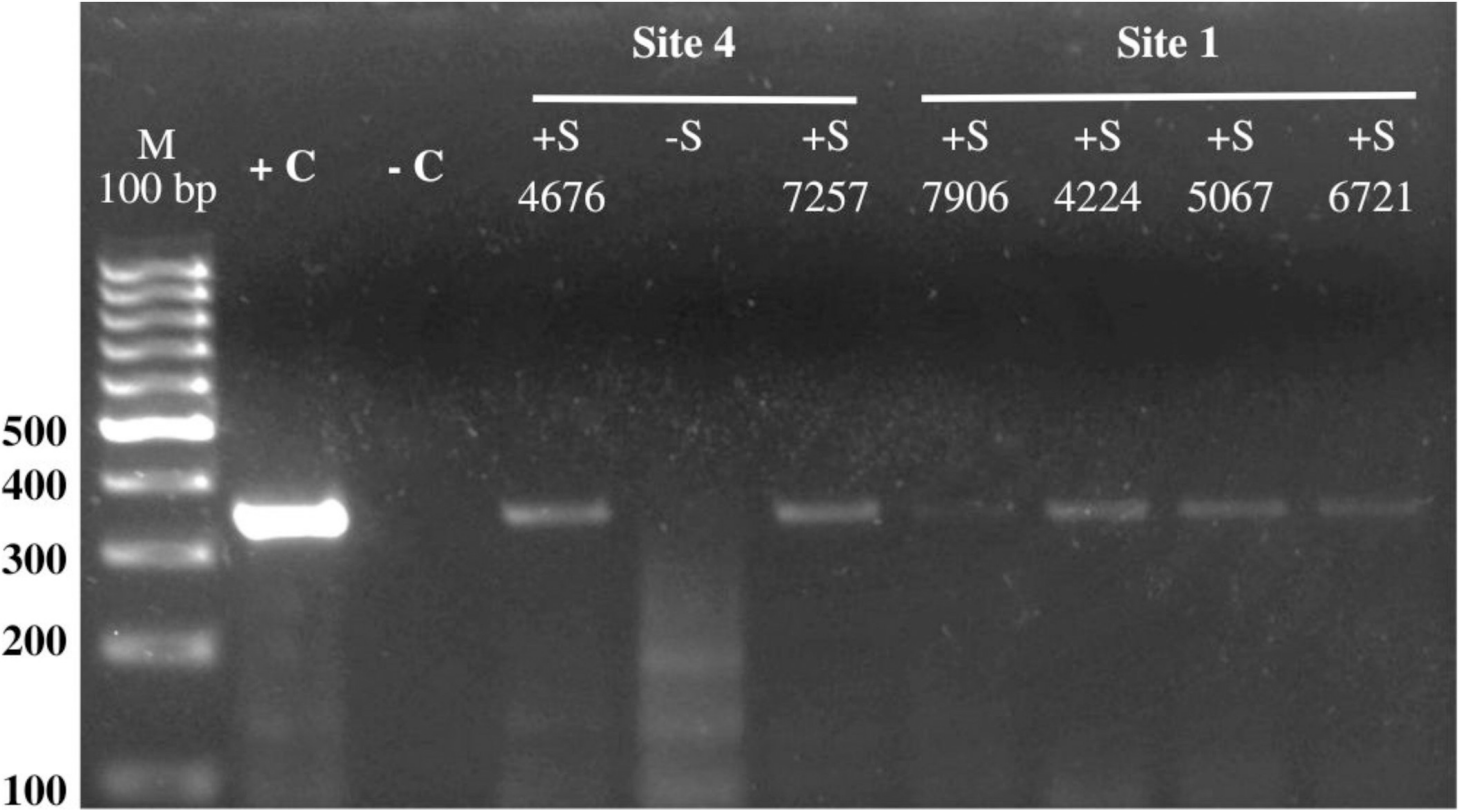
Result of the amplification of the ITS gene by PCR. Lane 1: 100 bp marker (Thermo Scientific), Lane 2: positive control (+C) corresponding to DNA from *L*. (*L*.) *mexicana* promastigotes in culture (strain: MHET/MX/97/Hd18); Lane 3: negative control (-C); Lane 4: *Leishmania* positive sample (+S) from site 4; Lane 5: *Leishmania* (-S) negative sample from site 4; Lane 6: *Leishmania* positive sample (+S) from site 4; Lanes 7–10: *Leishmania* (+S) positive samples from site 1.

An 18% (252/1404) of the collected *Pa*. *cratifer* females in Shannon Trap were analyzed for *Leishmania* detection. The percentage of these ranged between 12% and 80% (Table 2), by site. The examination revealed that in our study area, *L*. (*L*.) *mexicana* is the parasite species infecting *Pa*. *cratifer* females (Figs 4 and S2).

**Table 2:** The values in the first row represent the prevalence data of *L*. *(L*.*) mexicana* in *Pa*. *cratifer* per site (S1-S4). The total number of analyzed sandflies that were positive for *Leishmania* are represented in parenthesis. Values in brackets represented the 95% Confident Intervals. Months with different superscript letters (a-c) evidence significant differences in prevalence according to the paired comparison test using the Fisher’s exact test.

Site	% (n/N)	Nov	Dec	Jan	Feb	Mar	TOTAL
S1	12 (155/1252)	8 (3/37)^**a**^	6 (2/36)^**a**^	77 (23/30)^**b**^	83 (19/23)^**b**^	59 (17/29)^**b**^	41 (64/155)
		[1.7–21.9]	[0.6–18.6]	[57.7–90.0]	[61.2–95.0]	[38.9–76.3]	[35.9–0.520]
S2	80 (8/10)	-	0 (0/6)	-	0 (0/2)	-	0 (0/8)
		-	-	-	-	-	
S3	35 (13/37)	-	0 (0/6)	-	0 (0/7)	-	0 (0/13)
		-	-		-		
					30.3		
S4	72 (76/105)	0 (0/9)	0 (0/7)	42 (5/12)^**a**^	10 (2/21)^**a**^	41 (11/27)^**a**^	24 (18/76)^**a**^
		-	-	[15.1–72.3]	[1.1–30.3]	[22.3–61.2]	[14.6–34.8]
TOTAL	18 (252/1404)	7 (3/46)^**a**^	4(2/55)^**a**^	67 (28/42)^**b**^	40 (21/53)^**c**^	50 (28/56)^**b**^	33 (82/252)
		[1.3–17.8]	[0.4–12.5]	[55.4–84.2]	[26.4–53.9]	[36.3–63.6]	[26.7–38.7]

% = Percentage of individuals analyzed by site, N = Total number of females collected in Shannon Trap by site; n = Total number of *Pa*. *cratifer* females analyzed.

Our research revealed that in the transmission area of LCL, the prevalence of infection by *L*. *(L*.*) mexicana* in *Pa*. *cratifer* was of a 33% (CI = 26.7–38.7) (Table 2). In this study, the parasite prevalence of *Leishmania* in this sand fly species ranged between 6 and 83% (Table 2). All the positive samples for *L*. (*L*.) *mexicana* came from site 1 and site 4 (Table 2) and were not blood feed or gravid. Parasite prevalence values were significantly higher in S1 (41%) than in S4 (24%) (p = 0.008, Odd ration = 0.44) (Table 2).

Natural infection in females was registered through the transmission cycle of *L*. (*L*.) *mexicana*, which has been well-recognized in the Yucatan Peninsula. During the course of five months (November—March), the prevalence ranged between 4% and 67% (Table 2). Fisher’s Exact test results showed differences in the parasite prevalence between months (p = 0.0005). Parasite prevalence values were significantly higher in January (67%, CI = 55.4–84.2), March (50%, CI = 36.3–63.6), and February (40%, CI = 26.4–53.9). November and December recorded the lower prevalence with seven and four percent, respectively (Tables 2 and S4).

### Blood meal identification of *Pa*. *cratifer* females infected with *Leishmania*

**Fig 5:**
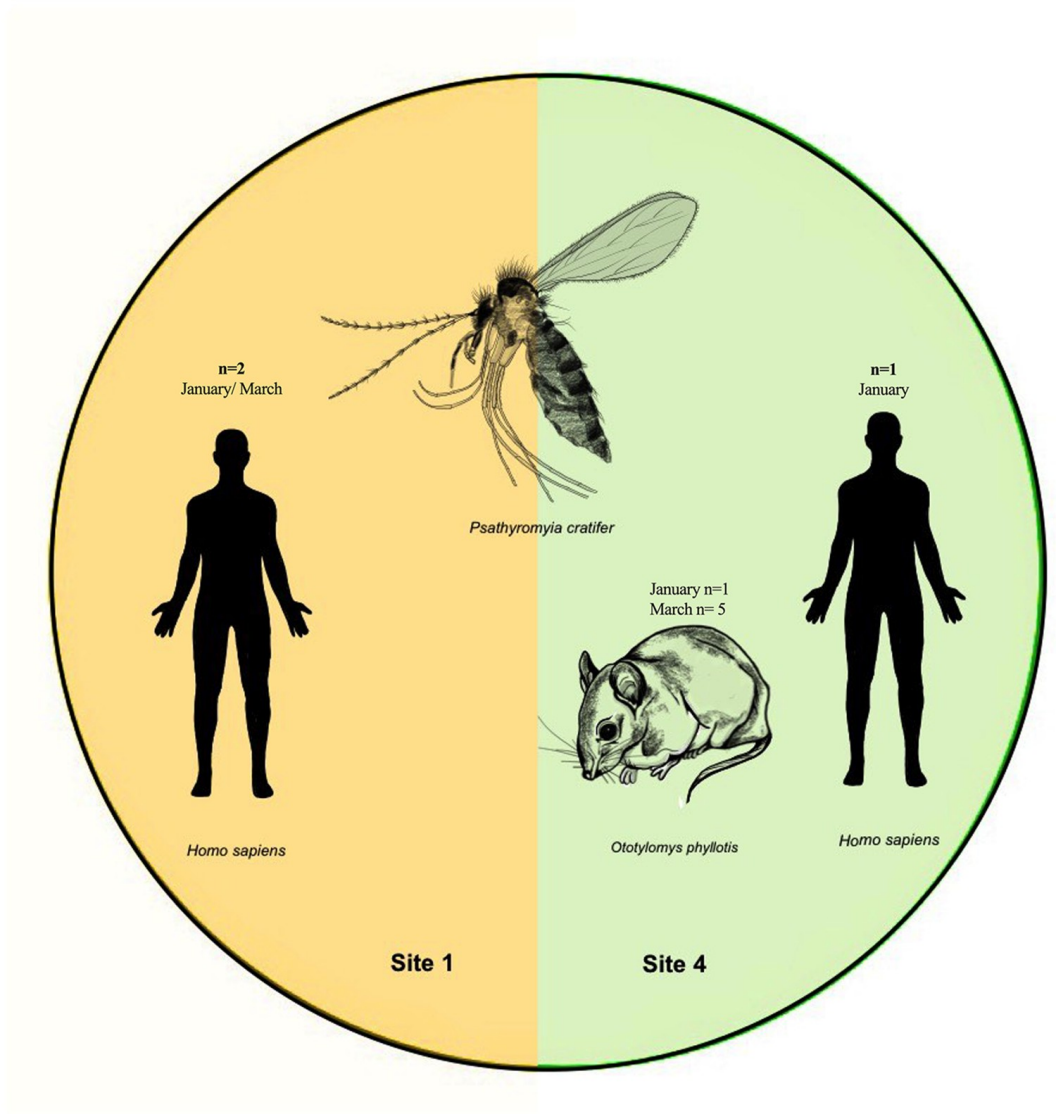
Blood meal sources detected by cytochrome b in individuals of *Pa*. *cratifer* infected with *Leishmania (L*.*) mexicana*. DNA samples (n = 20) from specimens collected in site 1 (n = 10) and site 4 (n = 10) were used to identify a portion of blood meals source (*Homo sapiens* and *Ototylomys phyllotis)*.

Ten DNA samples were analyzed from site 1 (Preserved tropical dry forest) and from site 4 (Secondary forest). Blood meal sources were successfully identified of nine individuals (45%) of *Pa*. *cratifer*. From these, two were collected from site 1 and seven from site 4 (S5 Table). Two blood host vertebrates were identified, *Ototylomys phyllotis* (78%) and *Homo sapiens* (22%) (Fig 5 and S5 Table). *Homo sapiens* was the only blood host detected in individuals of *Pa*. *cratifer* collected in S1 with Shannon trap. Both samples positive for human blood were collected during the months of highest prevalence of *Leishmania*, January and March. In site 4 most of the sequences identified (n = 6) corresponded to *O*. *phyllotis* (Fig 5 and S5 Table). In this site, *Homo sapiens* (n = 1) was identified in one sample collected in January while *O*. *phyllotis* was identified as a blood meal source in individuals collected in January (n = 1) and March (n = 5).

## Discussion

Our findings provide ecological and epidemiological evidence of the potential participation of *Pa*. *cratifer* in the transmission cycle of *L*. *mexicana* from an endemic area of LCL in Mexico. Using data from three different methods of collection, we documented: 1) a high abundance of females in the Shannon trap (1,404) when compared with CDC (88) and Disney (1) traps, which suggests the anthropophilic behavior of this sand fly species; 2) a temporal pattern of both abundance and *Leishmania* infection for *Pa*. *cratifer* within the well-recognized *Leishmania* transmission cycle in the YP, suggesting that its potential epidemiological risk may happen during the months of January through March; 3) the identification of *Homo sapiens* and *Ototylomys phyllotis* (a reservoir species previously documented in the region) as blood hosts of *Pa*. *cratifer*, which provides evidence about its potential role in the *L*. *mexicana* transmission cycle and 4) an hourly activity pattern in the first two hours of collection. Differences in the spatial distribution of *Leishmania* infection for *Pa*. *cratifer* were documented by using the Shannon trap in different collection sites from the emerging focus of LCL in Yucatan. Our data and those reported in a previous study [24] placed *Pa*. *cratifer* as one of the six potential vectors of *L*. *mexicana* in the YP. The findings incorporated in the present study contribute to the knowledge about elements involved in the potential transmission dynamics of LCL, and the potential development of control strategies within the region.

Nearly 32 years have passed since Professor Killick-Kendrick [58] published the major revision of the *Leishmania* vector incrimination criteria. Only about 10% of the approximately 1,020 species have been recognized as vectors [53,59]. In most of these sand fly species, not all incriminating criteria were covered in it for multiple reasons, mainly related to survival under laboratory conditions [60]. This work addresses the fact that *Pa*. *cratifer* satisfies three of the five most important previously established criteria in this LCL focus [60,61]. Firstly, there is a high abundancy of this species in the study area unlike others that have been previously documented in the region [24,28,29]. Secondly, the large number of specimens caught in Shannon trap and the blood meal data supported thar the females are anthropophilic and also showed the use of *O*. *phyllotis* reservoir as a blood host in the region. As a third point, females are infected with *L*. *(L*.*) mexicana* throughout the recognized transmission cycle in the region, a parasite species that has been identified as the causative agent of LCL in both humans [31], and wild reservoirs including *O*. *phyllotis* [41]. Even though the epidemiological and behavioral characteristics found in the present study suggest the potential participation of *Pa*. *cratifer* in the transmission of *L*. *mexicana*, our interpretation must be treated with caution until further studies demonstrate the ability of this species to support the full development of the parasite (i.e., examination of dissected females).

Previous to Montes de Oca-Aguilar et al. [24], several authors in Mexico did not consider *Pa*. *cratifer* as an anthropophilic species because its abundance ranged between 0.29%-3.3% in Shannon traps [18,19,28,36,37,38]. In spite of this, some undoubted vector populations appear to be small or have short longevity cycles that are potentially undetectable by sampling design [58]. When it comes about the low records of *Pa*. *cratifer* in the Disney trap it is not disregarded that it is due to the feeding of the wild hosts. The latter could be supported by the fact that so far, we have only detected the presence of *O*. *phyllotis* as a blood host, which is a dominant wild reservoir [41] in this LCL area, and not the *P*. *yucatanicus*.

Our results showed that the infection rate of *Pa*. *cratifer* is ranked similarly to the prevalence described in the proven vector *Brichomomyia olmeca olmeca* and *Lutzomyia cruciata* (Coquillett). Nonetheless it is high when compared with the values reported for *Psychodopygus panamensis* (Shannon), *Nyssomyia ylephiletor* (Fairchild and Hertig) and *Psathyromyia shannoni* (Dyar) [21,22,24]. For instance, *Bi*. *olmeca olmeca*, the only proven vector of *L*. *(L*.*) mexicana*, has documented infection rates of 1.5% and 33%, and the widely distributed and generalist *Lu*. *cruciata* recorded prevalences between 0.8% and 31%. Apropos, *Pa*. *shannoni*, a species that is phylogenetically close to *Pa*. *cratifer*, reports infection values between 5.4% (3/56) -15.1% (8/53) in the state of Campeche [21]. In Yucatan seven of the 16 specimens have tested positive for this *Leishmania* specie [25]. Unfortunately, the number of samples analyzed in these studies is very low, considering the number of specimens that could be captured in the sampling locations and this may underestimate the positive samples for *Leishmania* infection.

Our models demonstrated a marked difference in the abundance of *Pa*. *cratifer* between sites, fact that would be strongly linked to the characteristics of land use change and deforestation that occur along this LCL focus [29]. Regardless of this, the highest prevalence observed in *Pa*. *cratifer* matches with the high records in their natural landscape context (S1). Previously four sand flies species (*Bi*. *olmeca*, *Pa*. *shannoni*, *Lu*. *cruciata*, and *Lu*. *longipalpis*) of medical importance have been documented in S1. However, these exhibit low values of abundance compared to *Pa*. *cratifer* [24,29]. According to Sosa-Bibiano et al. [41], the S1 (Fragment of Tropical Dry Forest) reports only the presence of the wild reservoir *Heteromys gaumeri* with a high prevalence of *L*. *(L*.*) mexicana* (100%). Therefore, and considering the findings in this research, it is that we argue that the potential entomological risk from *Pa*. *cratifer* and the subsequent epidemiological risk for *Leishmania* are mainly sylvatic and enzootic, respectively. It is striking that the only two sites (S1 and S4) that register high abundances and infection rates in *Pa*. *cratifer* identify a strong dominance of the *H*. *gaumeri* reservoir. Despite this, our blood meal analysis only detects *O*. *phyllotis* as a blood host in one of the sites (S2) with low densities of *Pa*. *cratifer*. At the same time, infected individuals of *O*. *phyllotis* have been reported to be more abundant in S3 [41], other site with low densities and null prevalence of *Pa*. *cratifer*. Although more in-depth studies (spatial and temporal) on the blood meals of *Pa*. *cratifer* are still required, our findings show that this sand fly species makes use of one of the most important reservoirs of *Leishmania* in the region as blood host.

The evidence suggests that *Pa*. *cratifer* is a potentially vector of *L*. *(L*.*) mexicana* since it registers high infection rates throughout the enzootic cycle recognized to this pathogen in the YP. Interestingly, most of the females collected in Shannon Trap (98%) and all the samples that tested positive for *Leishmania* did not register a condition (i.e., fed, gravid) that would suggest previous blood intake. This proposes that the *Leishmania* parasite persists still even after the apparent excretion of the digested blood meal. Detection of infection in females that do not contain recent blood meals is an essential element to incriminate a species [59], which reinforces our argument that *Pa*. *cratifer* would be a competent vector. On the other hand, our data showed that regardless of the site, the period of highest entomological risk to *Pa*. *cratifer* occurs from January to March, which matches with the high infection rates by *L*. *(L*.*) mexicana*. In addition, it is worth noting that our results evidenced a temporal pattern of prevalence different from that registered in other endemic foci of *L*. *(L*.*) mexicana* in the YP. The latter, since so far, the highest infection rates have been documented in the months of November and December [21,23]. Although in Yucatan the authors Pérez-Blas et al. [25] documented sand flies infected with *L*. *(L*.*) mexicana* in February, their results cannot be compared with our findings since the prevalence values were estimated only for specimens collected in that period. In that sense our sampling period, which is based on the recognized transmission cycle for the parasite in the region, is likely to be a partial reflection of the period of activity and infection rates of *Pa*. *cratifer*. For this reason, it is important to conduct studies with longer time intervals (i.e., annual or stational) to generate information on the bionomics and temporal distribution trends of this sand fly species.

Temperature and humidity are factors that are strongly related to the survival and development time of sand fly species and the transmission of LCL [62,63]. However, our findings showed that both variables do not modulate *Pa*. *cratifer* population dynamics as previously documented in the region to *Lu*. *cruciata*, *Psychopygus panamensis*, and the *Bi*. *olmeca olmeca* [19, 64]. Nevertheless, the results shown herein are based on four sites where the highest period of activity was detected. There, the infection rate of this potential *Leishmania* vector begins with the decline of the coldest season (November-February) and the beginning of the driest period in the region (March-June) [39]. There are other environmental (precipitation) or ecological (host distribution and abundance) variables that were not considered in our study that may be modulating the population dynamics of *Pa*. *cratifer*.

Yet, our findings reinforce the need to develop further studies that consider longer time intervals (i.e., stational) to determine the effect of ecological variables on the abundance of this species. The hourly activity pattern observed in *Pa*. *cratifer* females is similar to that observed in other *L*. *mexicana* vector species in the region [21,22]. Notwithstanding the preceding, it seems that the forest conservation status of each site may be influencing the behavior pattern, as observed in sites 2 and 3 (S2 Fig).

In conclusion, our results show the relevance of directing the attention to *Pa*. *cratifer* and considering it as a potential vector for *L*. *(L*.*) mexicana* in southern Mexico. The geographic distribution of *Pa*. *cratifer* within the country has yet to be fully known, so it is still possible to associate it with other areas of autochthonous and active transmission of LCL. The degree of participation of *Pa*. *cratifer* in the emergence of LCL in various regions of Yucatan needs to be resolved. Additionally, our findings also evidence that even in southern Mexico (which represents an area with a significant and historical solid contribution to the study of *Leishmania* vectors) entomological research on LCL is still scarce. Also, it seems that parasite-vector interactions could be even more complex. With these results, it is possible to argue that *Pa*. *cratifer* is the sixth species of sand fly that is potentially involved in the transmission cycles of *L*. *mexicana* in southern Mexico. Several aspects of the biology, behavior, and ecology of such and other species of sand flies need to be addressed along with other fundamental elements. The latter, to be able to predict the risk of transmission and expansion of leishmaniasis in the YP and other regions of the country. At the same time, we believe it is crucial to conduct studies in other Central American countries where *Pa*. *cratifer* also happens, and where the prevalence of *L*. (*L*.) *mexicana* is greater.

## Supporting information

S1 Table Results of the ANOVA of models on differences in temperature (normal distribution, T°C) and relative humidity (beta distribution, RH) in fourth sites in southern Mexico. (DOCX)

S2 Table Results of the analysis of variance on a generalized linear model with negative binomial distribution where abundance was considered as a dependent variable, and month, sex and their interaction as independent variables. Significant P values are in boldface (p<0.005).
(DOCX)

S3 Table General mixed model result using a Negative binomial distribution where abundance was considered as dependent variable, and temperature (°C) and relative humidity (HR, %) as independent variables. Significant P values are in boldface (p<0.005).
(DOCX)

S4 Table Results of the comparison of the prevalence of *Leishmania* infection in *Pa*. *cratifer* between months according to the Fisher´s exact test. (DOCX)

S5 Table Blood meal sources detected by cytochrome b in individuals of *Pa*. *cratifer*. (DOCX)

S1 Fig Temporal variation in the hourly activity of *Pa*. *cratifer* in four sites of an emerging focus of cutaneous leishmaniasis in Yucatan, Mexico. Site 1 (S1), Site 2 (S2), Site 3 (S3), and Site 4 (S4).
(DOCX)

S2 Fig Representative gel of PCR-RFLP after *Hae* III digestion of a 300–350 bp ITS-1 DNA fragment obtained with fourth specimens of *Pa*. *cratifer* lanes l-4: Restriction digestion profiles of fourth specimens of *Pa*. *cratifer*. Lanes: L. m. (*L*. *mexicana* (MHOM/MX/2011/Lacandona)), L. a. (*L*. *amazonensis* (MHOM/BR/1973/M2269)), L. b. (*L*. *braziliensis* (MHOM/BR/1995/M15280)), and L. i. (*L*. *infantum* (MHOM/BR/72/BH46)) were used as positive controls. lane neg: negative control. M: molecular marker (25 bp). (DOCX)
