# Supplementary material for: Abundance and Leishmania infection patterns of the sand fly Psathyromyia cratifer in Southern Mexico
Source: PLoS Negl Trop Dis. 2024 Sep 10;18(9):e0012426. doi: 10.1371/journal.pntd.0012426 (PMC11414901; doi:10.1371/journal.pntd.0012426)
Supplement: S1 Table — (DOCX) [file pntd.0012426.s001.docx]

**S1 Table.** Results of the ANOVA of models on differences in temperature (normal distribution, T°C) and relative humidity (beta distribution, RH) in fourth sites in southern Mexico

| T °C | **df** | **F value** | **Pr(>F)** | **R^2^** |  | RH | **df** | **Chisq** | **Pr(>Chisq)** | **PseudoR^2^** |
| --- | --- | --- | --- | --- | --- | --- | --- | --- | --- | --- |
| Site | 3 | 6.53 | 0.00 | 40.1 |  | Site | 3 | 21.44 | 0.00 | 51 |
| Month | 4 | 55.30 | < 2.2e-16 |  |  | Month | 4 | 299.86 | < 2.2e-16 |  |
| Site:Month | 12 | 9.54 | < 2.2e-16 |  |  | Site:Month | 12 | 103.00 | < 2.2e-16 |  |
