# Supplementary material for: Abundance and Leishmania infection patterns of the sand fly Psathyromyia cratifer in Southern Mexico
Source: PLoS Negl Trop Dis. 2024 Sep 10;18(9):e0012426. doi: 10.1371/journal.pntd.0012426 (PMC11414901; doi:10.1371/journal.pntd.0012426)
Supplement: S2 Table — Significant P values are in boldface (p<0.005). (DOCX) [file pntd.0012426.s002.docx]

**S2 Table.** Results of the analysis of variance on a generalized linear model with negative binomial distribution where abundance was considered as a dependent variable, and month, sex and their interaction as independent variables. Significant P values are in boldface (p<0.005).

| **Site** | **Component** | **χ^2^** | **Df** | **Pr(>Chisq)** |
| --- | --- | --- | --- | --- |
| S1 | Month | 30.2599 | 4 | 4.33e-06 |
|  | Sex | 1.0112 | 1 | 0.3146 |
|  | Month:Sex | 3.0892 | 4 | 0.543 |
|  |  |  |  |  |
| S2 | Month | 5.3634 | 4 | 0.252 |
|  | Sex | 0.4976 | 1 | 0.4806 |
|  | Month:Sex | 0.2337 | 4 | 0.9937 |
|  |  |  |  |  |
| S3 | Month | 7.0245 | 4 | < 2.2e-16 |
|  | Sex | 1.0011 | 1 | 0.32 |
|  | Month:Sex | 1.0949 | 4 | 0.8951 |
|  |  |  |  |  |
| S4 | Month | 10.9561 | 4 | 0.2706 |
|  | Sex | 1.392 | 1 | 0.23808 |
|  | Month:Sex | 1.0900 | 4 | 0.8700 |
