# Supplementary material for: Abundance and Leishmania infection patterns of the sand fly Psathyromyia cratifer in Southern Mexico
Source: PLoS Negl Trop Dis. 2024 Sep 10;18(9):e0012426. doi: 10.1371/journal.pntd.0012426 (PMC11414901; doi:10.1371/journal.pntd.0012426)
Supplement: S3 Table — Significant P values are in boldface (p<0.005). (DOCX) [file pntd.0012426.s003.docx]

**S3 Table.** General mixed model result using a Negative binomial distribution where abundance was considered as dependent variable, and temperature (°C) and relative humidity (HR, %) as independent variables. Significant P values are in boldface (p<0.005).

| **Response variable** | **Component** | **Estimate** | **Standard Error** | **z** | **P value** | **AIC** | **Deviance** |
| --- | --- | --- | --- | --- | --- | --- | --- |
|  | Null Model | 4.28 | 0.40 | 10.68 | <2e-16 | 193 | 0 |
|  |  |  |  |  |  |  |  |
| Total_abundance_ | Intercept | 3.33 | 2.87 | 1.16 | 0.25 | 194 | 0 |
|  | ~RH | 0.01 | 0.03 | 0.33 | 0.74 |  |  |
|  |  |  |  |  |  |  |  |
|  | Intercept | 6.17 | 4.80 | 1.29 | 0.20 | 194 | 0.6 |
|  | ~Temp (°C) | -0.08 | 0.20 | -0.40 | 0.69 |  |  |
|  |  |  |  |  |  |  |  |
| S1_Abundance_ | Intercept | 4.49171 | 1.24731 | 3.601 | **0.00** | 399 | 1 |
|  | ~Temp (°C) | -0.04647 | 0.05062 | -0.918 | 0.36 |  |  |
|  |  |  |  |  |  |  |  |
|  | Intercept | 4.35391 | 1.03943 | 4.189 | **0.00** | 399 | 1 |
|  | ~RH | -0.01148 | 0.01176 | -0.976 | 0.33 |  |  |
|  |  |  |  |  |  |  |  |
| S2_Abundance_ | Intercept | -1.116807 | 3.127272 | -0.357 | 0.72 | 65 | 0.01 |
|  | ~Temp (°C) | -0.005169 | 0.128963 | -0.04 | 0.97 |  |  |
|  |  |  |  |  |  |  |  |
|  | Intercept | -7.73827 | 3.64314 | -2.124 | **0.03** | 60 | 17 |
|  | ~RH | 0.07025 | 0.03795 | 1.851 | 0.06 |  |  |
|  |  |  |  |  |  |  |  |
| S3 _Abundance_ | Intercept | -1.26215 | 2.75944 | -0.457 | 0.65 | 116 | 0.01 |
|  | ~Temp (°C) | 0.04377 | 0.11562 | 0.379 | 0.71 |  |  |
|  |  |  |  |  |  |  |  |
|  | Intercept | 3.52451 | 1.33459 | 2.641 | **0.01** | 111 | 13 |
|  | ~RH | -0.04668 | 0.01656 | -2.819 | 0.051 |  |  |
|  |  |  |  |  |  |  |  |
| S4 _Abundance_ | Intercept | 2.25446 | 1.85347 | 1.216 | 0.22 | 179 | 0.1 |
|  | ~Temp (°C) | -0.06201 | 0.07607 | -0.815 | 0.42 |  |  |
|  |  |  |  |  |  |  |  |
|  | Intercept | 3.77031 | 1.34782 | 2.797 | **0.01** | 175.68 | 9 |
|  | ~RH | -0.03697 | 0.01611 | -2.295 | 0.061 |  |  |
