# Supplementary material for: Abundance and Leishmania infection patterns of the sand fly Psathyromyia cratifer in Southern Mexico
Source: PLoS Negl Trop Dis. 2024 Sep 10;18(9):e0012426. doi: 10.1371/journal.pntd.0012426 (PMC11414901; doi:10.1371/journal.pntd.0012426)
Supplement: S4 Table — (DOCX) [file pntd.0012426.s004.docx]

**S4 Table.** Results of the comparison of the prevalence of *Leishmania* infection in *Pa. cratifer* between months according to the Fisher´s exact test.

| **Component** | **Month** | **Comparison p Fisher** | **P adj Fisher** |
| --- | --- | --- | --- |
| General | Nov - Dic | 1.00 | 1.00 |
|  | Nov - Jan | 0.00 | **0.000** |
|  | Nov - Feb | 0.00 | **0.000** |
|  | Nov - Mar | 0.00 | **0.000** |
|  | Dic - Jan | 0.00 | **0.000** |
|  | Dic - Feb | 0.00 | **0.000** |
|  | Dic - Mar | 0.00 | **0.000** |
|  | Jan - Feb | 0.01 | **0.018** |
|  | Jan - Mar | 0.15 | 0.185 |
|  | Feb - Mar | 0.34 | 0.374 |
| S1 | Nov - Dic | 1.00 | 1.00 |
|  | Nov - Jan | 0.00 | **0.00** |
|  | Nov - Feb | 0.00 | **0.00** |
|  | Nov - Mar | 0.00 | **0.00** |
|  | Dic - Jan | 0.00 | **0.00** |
|  | Dic - Feb | 0.00 | **0.00** |
|  | Dic - Mar | 0.00 | **0.00** |
|  | Jan - Feb | 0.74 | 0.82 |
|  | Jan - Mar | 0.17 | 0.21 |
|  | Feb - Mar | 0.08 | 0.11 |
| S2 | Jan - Feb | 0.412 | 0.412 |
|  | Jan - Mar | 0.229 | 0.344 |
|  | Feb - Mar | 0.0222 | 0.066 |
