# Supplementary material for: Abundance and Leishmania infection patterns of the sand fly Psathyromyia cratifer in Southern Mexico
Source: PLoS Negl Trop Dis. 2024 Sep 10;18(9):e0012426. doi: 10.1371/journal.pntd.0012426 (PMC11414901; doi:10.1371/journal.pntd.0012426)
Supplement: S5 Table — (DOCX) [file pntd.0012426.s005.docx]

**S5 Table.** Blood meal sources detected by cytochrome b in individuals of *Pa. cratifer*.

| **ID** | **Site** | **Month** | **Identities (%)** | **Accession number**  **(Host)** | **Blood meal source** | **ID Submittion Genbank** (Provisional) |
| --- | --- | --- | --- | --- | --- | --- |
| 5067 | Preserved tropical dry forest (S1) | January | 273/294 (93%) | MH444420.1 | *Homo sapiens* | PP923724 |
| 7906 | Preserved tropical dry forest (S1) | March | 275/279 (99%) | KX4570031.1 | *Homo sapiens* | PP923725 |
| 4776 | Secondary forest (S2) | January | 285/289 (99%) | KJ751498.2 | *Ototylomys* sp. | PP923726 |
| 4800 | Secondary forest (S2) | January | 271/293 (93%) | OQ858944.1 | *Homo sapiens* | PP923723 |
| 7257 | Secondary forest (S2) | March | 289/290 (99%) | JX020638.1 | *Ototylomys phyllotis* | PP923729 |
| 7267 | Secondary forest (S2) | March | 297/300 (99%) | JX020638.1 | *Ototylomys phyllotis* | PP923730 |
| 7237 | Secondary forest (S2) | March | 295/297 (99%) | JX020638.1 | *Ototylomys phyllotis* | PP923727 |
| 7240 | Secondary forest (S2) | March | 293/296 (99%) | JX020638.1 | *Ototylomys phyllotis* | PP923728 |
| 7272 | Secondary forest (S2) | March | 288/292 (99%) | JX020638.1 | *Ototylomys phyllotis* | PP923731 |
