# Supplementary material for: Abundance and Leishmania infection patterns of the sand fly Psathyromyia cratifer in Southern Mexico
Source: PLoS Negl Trop Dis. 2024 Sep 10;18(9):e0012426. doi: 10.1371/journal.pntd.0012426 (PMC11414901; doi:10.1371/journal.pntd.0012426)
Supplement: S1 Fig — Site 1 (S1), Site 2 (S2), Site 3 (S3), and Site 4 (S4). (DOCX) [file pntd.0012426.s006.docx]

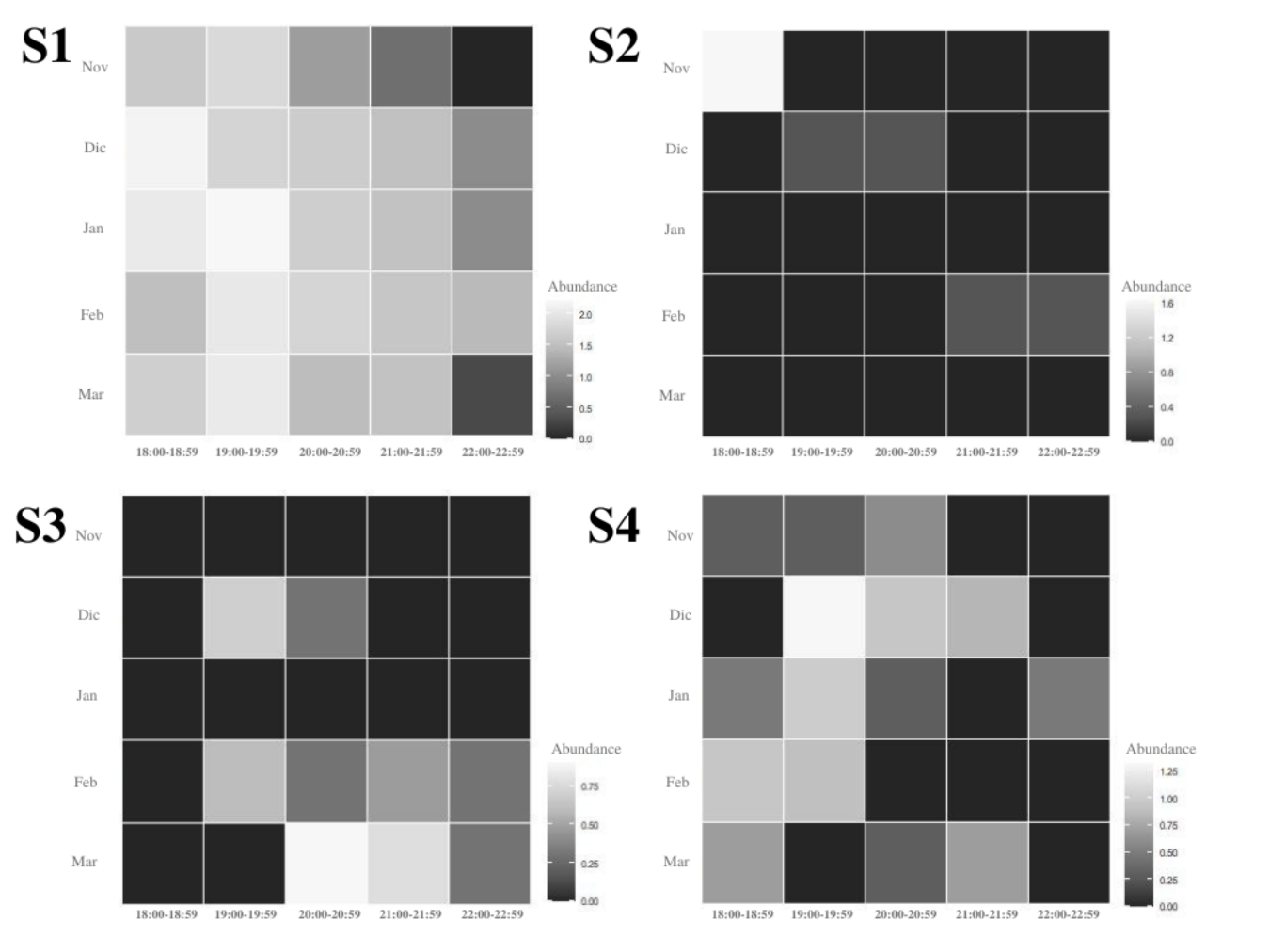


**S1 Fig.** Temporal variation in the hourly activity of *Pa. cratifer* in four sites of an emerging focus of cutaneous leishmaniasis in Yucatan, Mexico. Site 1 (S1), Site 2 (S2), Site 3 (S3), and Site 4 (S4).
