# Supplementary material for: Abundance and Leishmania infection patterns of the sand fly Psathyromyia cratifer in Southern Mexico
Source: PLoS Negl Trop Dis. 2024 Sep 10;18(9):e0012426. doi: 10.1371/journal.pntd.0012426 (PMC11414901; doi:10.1371/journal.pntd.0012426)
Supplement: S2 Fig — (DOCX) [file pntd.0012426.s007.docx]

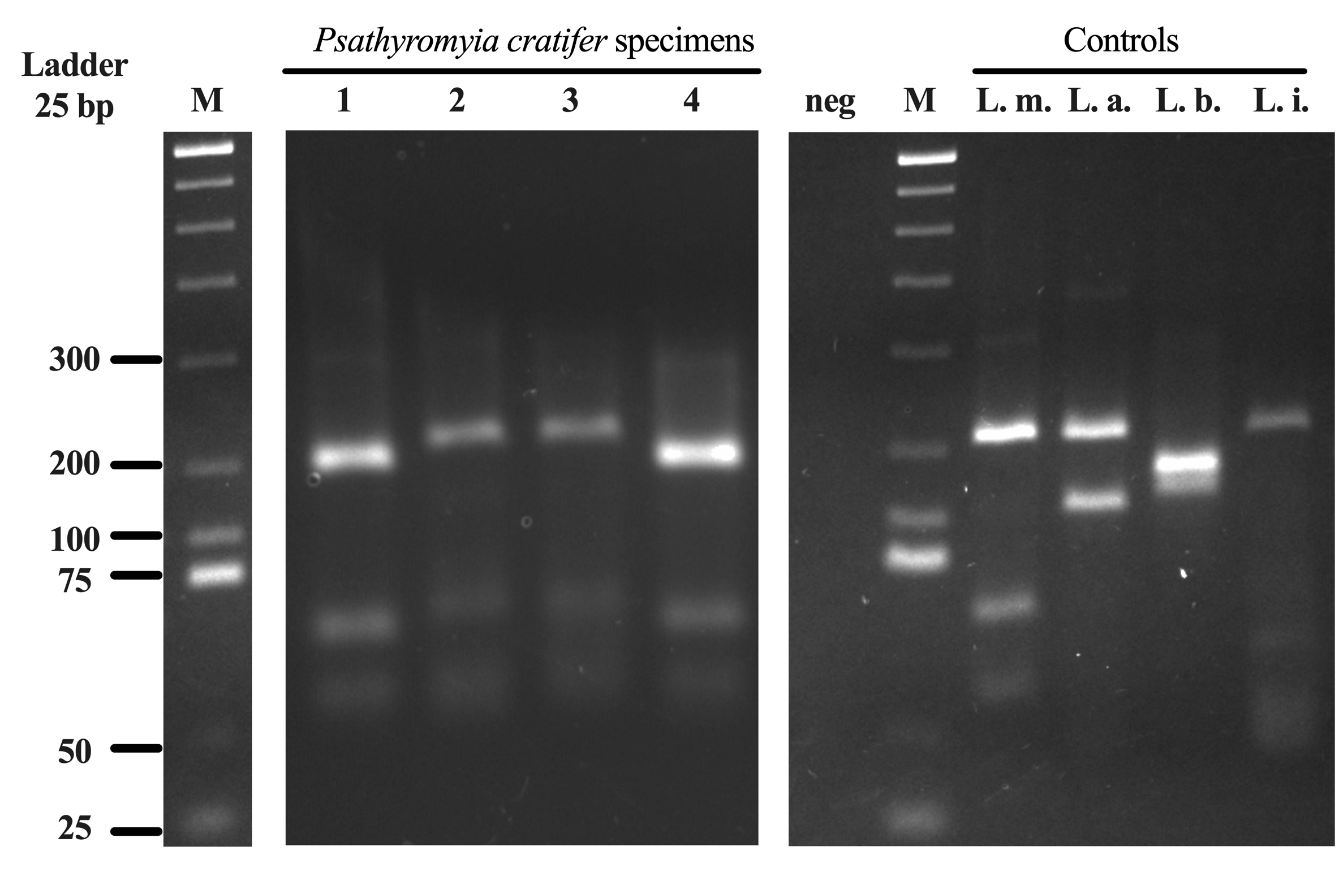


**S2 Fig.** Representative gel of PCR-RFLP after *Hae* III digestion of a 300-350 bp ITS-1 DNA fragment obtained with fourth specimens of *Pa. cratifer* lanes l-4: Restriction digestion profiles of fourth specimens of *Pa. cratifer*. Lanes: L. m. (*L. mexicana* (MHOM/MX/2011/Lacandona)), L. a. (*L. amazonensis* (MHOM/BR/1973/M2269)), L. b. (*L. braziliensis* (MHOM/BR/1995/M15280)), and L. i. (*L. infantum* (MHOM/BR/72/BH46)) were used as positive controls. lane neg: negative control. M: molecular marker (25 bp).
